# Supplementary material for: Topological magneto-optical effect from skyrmion lattice
Source: Nat Commun. 2023 Sep 5;14:5416. doi: 10.1038/s41467-023-41203-y (PMC10480175; doi:10.1038/s41467-023-41203-y)
Supplement: Supplementary file 1 — Supplementary information [file 41467_2023_41203_MOESM1_ESM.pdf]

Supplemental information for "Topological magneto-optical effect from skyrmion lattice"

Yoshihiro D. Kato[1], Yoshihiro Okamura[1*], Max Hirschberger[1,2],

Yoshinori Tokura[1,2,3], and Youtarou Takahashi[1,2*]

[1]*Department of Applied Physics and Quantum Phase Electronics Center, University of Tokyo, Tokyo 113-8656, Japan*

[2]*RIKEN Center for Emergent Matter Science (CEMS), Wako 351-0198, Japan*

[3]*Tokyo College, University of Tokyo, Tokyo 113-8656, Japan*

[*]To whom correspondence should be addressed (okamura@ap.t.u-tokyo.ac.jp, youtarou-takahashi@ap.t.u-tokyo.ac.jp).

Supplementary Note 1: Contribution of the normal Hall effect and intraband transition related to the real-space Berry phase to the optical Hall conductivity.

We evaluate the contribution of the normal Hall effect to the optical Hall conductivity spectra, which is not considered in deducing the topological Hall conductivity spectra. In the Drude model, $\sigma_{xy}(\omega)$ from the normal Hall effect is expressed as

$$\sigma_{xy}(\omega) = \frac{ne^2}{m^*}\frac{\omega_c}{\left(\omega + \frac{i}{\tau}\right)^2 - \omega_c^2}, \tag{S1}$$

where n is the carrier density, m^* is the effective mass, τ is the scattering time, and $\omega_c = eB/m^*$ is the cyclotron frequency. Supplementary Figure 6 shows the $\sigma_{xy}(\omega)$ spectra calculated from Eq. (S1) at 0.49 T with using $n = 7.8 \times 10^{21}$ cm^{-3}, $m^* = 7.4m_e$ (m_e: electron mass), and $\frac{\hbar}{\tau} = 15$ meV. These parameters are determined from transport properties[1] and the Drude response observed in the optical conductivity (Supplementary Fig. 5). The calculated optical Hall conductivity shows a resonance structure below 20 meV. We found that, above 40 meV, the magnitude of the resonance is negligibly small as compared to the observed $\sigma_{xy}^{\mathrm{T}}(\omega)$ and $\sigma_{xy}^{\mathrm{M}}(\omega)$ (Supplementary Fig. 6). In addition, even below 20 meV, $\sigma_{xy}^{\mathrm{T}}(\omega)$ and $\sigma_{xy}^{\mathrm{M}}(\omega)$ are dominant for the optical Hall response, consistent with the fact that the magnitude of the d.c. topological and anomalous Hall conductivities is much larger than that of the d.c. normal Hall conductivity. Therefore, we omit the contribution from the normal Hall effect in the analysis of optical spectra.

In addition, the real-space Berry phase results in the real-space emergent magnetic field, which induces the electron's traverse motion and topological Hall effect. Since this picture is analogous to the normal Hall effect induced by the Lorentz force, the

topological optical Hall conductivity related to the real-space Berry phase can be described by the Drude model under the emergent magnetic field B_{eff}. Accordingly, this intraband contribution $\sigma_{xy}^{\mathrm{T,\ intra}}(\omega)$ is expressed as,

$$\sigma_{xy}^{\mathrm{T,\ intra}}(\omega) = \frac{ne^2}{m^*} \frac{\omega_c^{eff}}{\left(\omega + \frac{i}{\tau}\right)^2 - \omega_c^{eff^2}}, \tag{S2}$$

where n is the carrier density, m^* is the effective mass, τ is the scattering time, and $\omega_c^{eff} = eB_{eff}/m^*$ is the cyclotron frequency. Here, we put $B_{eff} = 23.5$ T. To see this spectral response, we assume that the difference between $\sigma_{xy}^{\mathrm{T}}(\omega = 0 \text{ meV})$ and $\sigma_{xy}^{\mathrm{T}}(\omega = 40 \text{ meV})$ comes entirely from the intraband contribution. Supplementary Figure 7 shows the resultant $\sigma_{xy}^{\mathrm{T,\ intra}}(\omega)$ spectrum at 0.49 T. We find that the intraband contribution is steeply enhanced below the present energy window (~ 40 meV) and dominates the $\sigma_{xy}(\omega)$ response below 30 meV.

Supplementary Note 2: The effect of extrapolation of the magneto-optical spectra below 40 meV.

In the present Kramers-Kronig analysis, we assume the ω-linear function for the low-energy extrapolation, which is verified in the metallic sample as discussed in the following. In the low-energy region, for example, below 30 or 40 meV, $\varepsilon_{xx}(\omega)$ is dominated by the Drude response and shows the $1/\omega^2$ divergence. Therefore, the Kerr rotation and ellipticity, which are given by $-\frac{\sigma_{xy}(\omega)}{\sigma_{xx}(\omega)\sqrt{\varepsilon_{xx}(\omega)}}$ and inversely proportional to square root of $\varepsilon_{xx}(\omega)$, should be roughly proportional to ω, if we assume the Hall angle $\sigma_{xy}(\omega)/\sigma_{xx}(\omega)$ is constant. We also test several other extrapolation functions described by $\omega^{1/3}$, $\omega^{1/2}$, ω^2, and ω^3 as shown in Supplementary Fig. 10a. The resultant Re $\sigma_{xy}(\omega)$

spectra tend to depend on the assumed functions at the lowest energy, but the spectral characteristics in the discussed energy range above 0.04 eV are robustly unchanged. Meanwhile, the Im $\sigma_{xy}(\omega)$ spectra are hardly affected by the interpolation (Supplementary Fig. 10b). Thus, the choice of the interpolation functions does not affect the conclusions and discussions in the main text.

a b

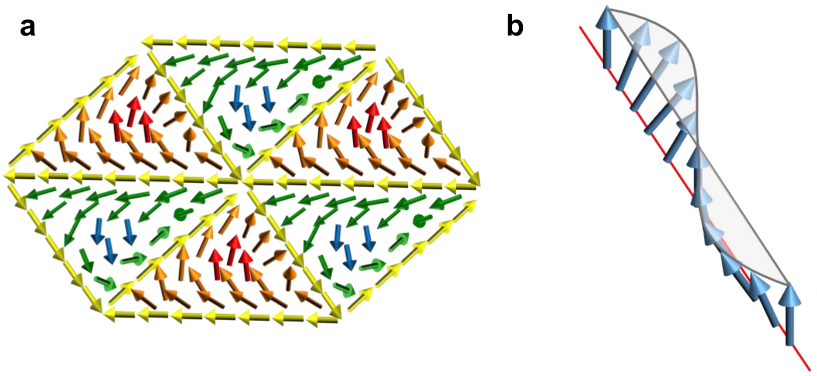

Supplementary Figure 1| Spin structures for IC-1 and IC-2 phases. a, A schematic illustration of the putative spin structure for the IC-1 phase (meron-antimeron structure)[2,3]. **b**, A schematic illustration of the spin structure in the IC-2 phase (fan-like structure)[2,3].

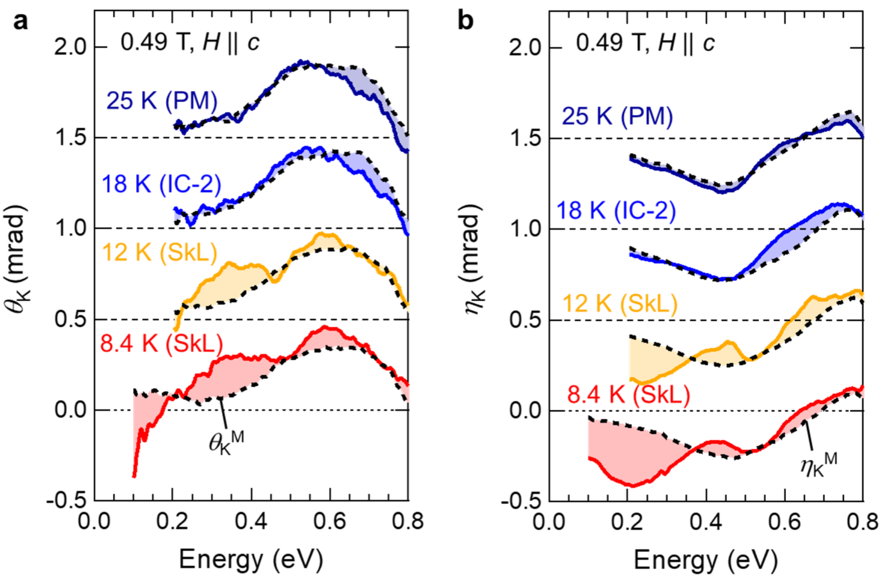

Supplementary Figure 2| Temperature dependence of magneto-optical Kerr effect.

Temperature dependence of magneto-optical Kerr (a) rotation angle and (b) ellipticity for $H \parallel c$ at 0.49 T. The data are shifted by vertical offsets of 0.5 mrad. The black dotted curves denote the conventional M-linear MOKE θ_K^M and η_K^M; the shaded areas represent the topological components θ_K^T and η_K^T.

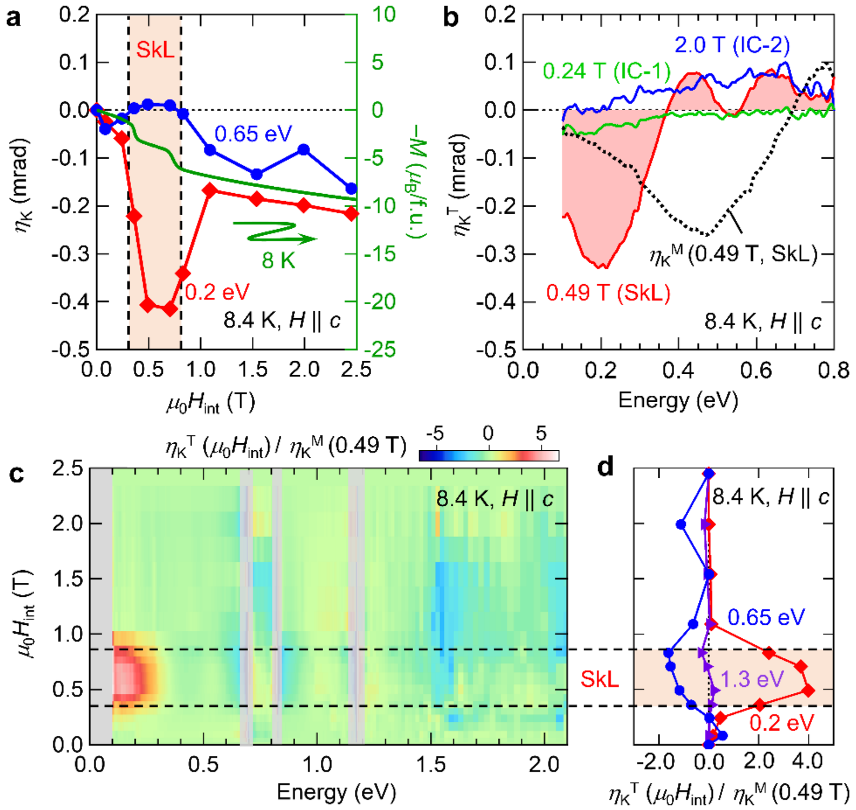

Supplementary Figure 3| Topological component of the magneto-optical Kerr ellipticity. a, Magnetic-field dependence of the Kerr ellipticity η_K at 0.2 eV (red) and at 0.65 eV (blue) at 8.4 K, and of the magnetization M (green curve) at 8 K. The dashed vertical lines represent the phase boundaries of the SkL phase. **b**, Topological component of the Kerr ellipticity η_K^T at 0.24 T (IC-1 phase, green curve), at 0.49 T (SkL phase, red curve), and at 2.0 T (IC-2 phase, blue curve). The dotted curve represents the conventional M-induced component in the SkL phase (0.49 T), η_K^M(0.49 T). Here, η_K^T and η_K^M are defined in the same manner as θ_K^T and θ_K^M: $\eta_K^T(\omega) = \eta_K(\omega) - \eta_K^M(\omega) = \eta_K(\omega) - \frac{M(\mu_0 H_{int})}{M(2.5\ T)}\eta_K(\omega, 2.5\ T)$. **c**, Magnetic-field dependence of $\eta_K^T(\mu_0 H_{int})$ normalized by η_K^M(0.49 T). As η_K^M(0.49 T) traverses zero around 0.7 eV, 0.8 eV, and 1.15 eV, $\eta_K^T(\mu_0 H_{int})/\eta_K^M$(0.49 T) around these energies tend to diverge in the whole magnetic-

field region. The corresponding data are covered by the transparent gray bars. **d**, Magnetic-field dependence of $\eta_K^T(\mu_0 H_{int})/\eta_K^M(0.49\ T)$ for 0.2 eV (red), 0.65 eV (blue), and 1.3 eV (purple). In **c** and **d**, the dotted horizontal lines represent the phase boundaries of the SkL phase.

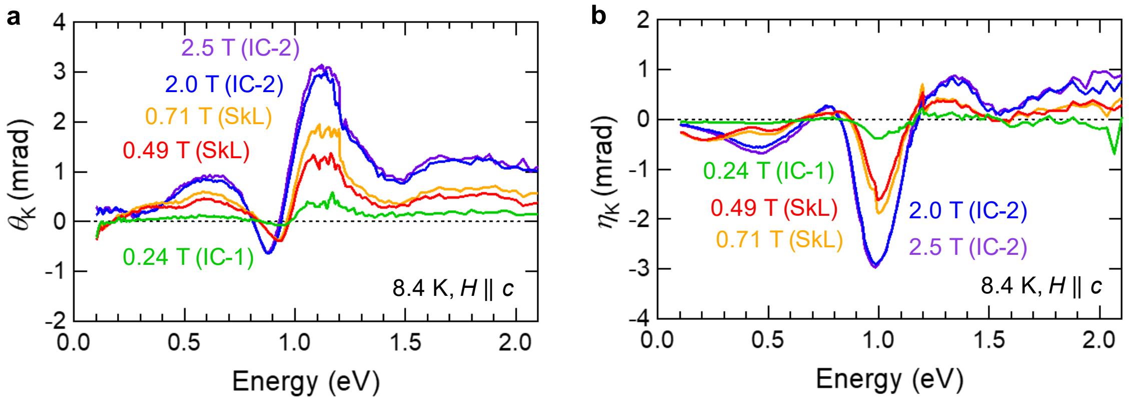

Supplementary Figure 4| MOKE spectra over a wide energy range. a,b, Magnetic-field dependence of magneto-optical Kerr (**a**) rotation angle θ_K and (**b**) ellipticity η_K for $H \parallel c$ at 8.4 K from 0.1 to 2.1 eV.

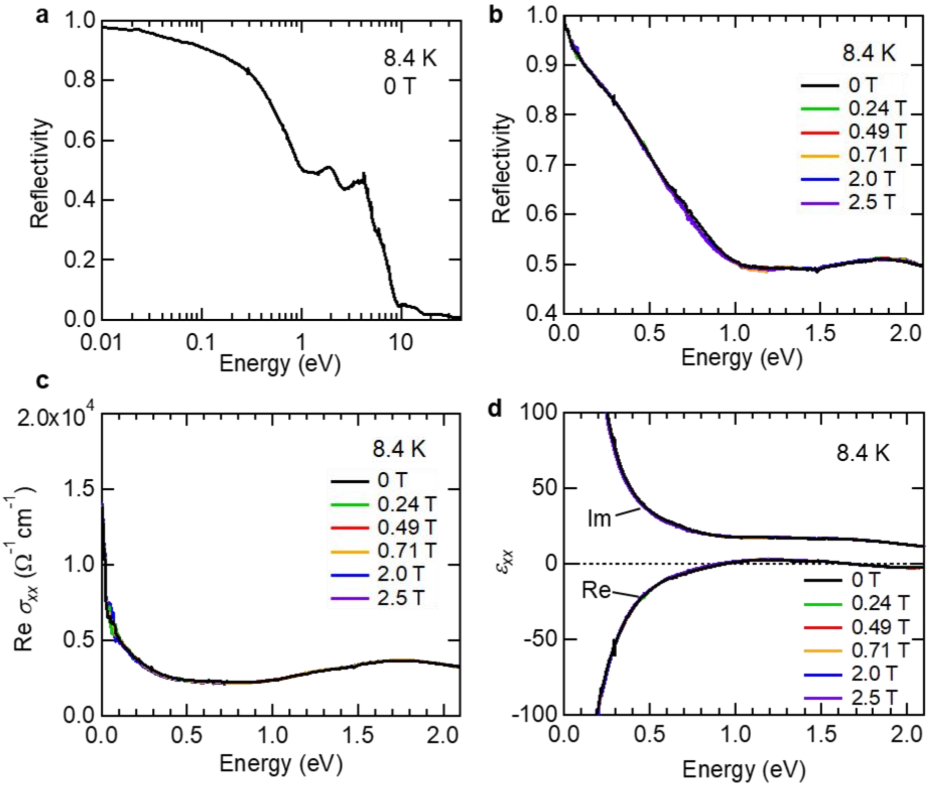

Supplementary Figure 5| Optical conductivity and dielectric constant obtained from the reflectivity spectra at 8.4 K. a, Reflectivity spectrum at 0 T. **b-d**, Magnetic-field dependence of (**b**) reflectivity, (**c**) real part of optical conductivity Re σ_{xx}, and (**d**) diagonal component of dielectric constant ε_{xx}. Optical conductivity and dielectric constant were deduced from a Kramers-Kronig transformation of the reflectivity spectra. Within the present experiment, no discernible spectral change is observed by the application of a magnetic field.

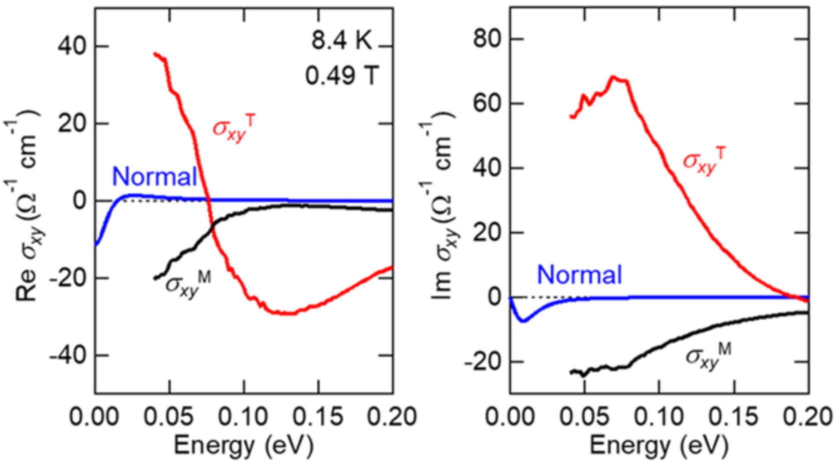

Supplementary Figure 6| Contribution from normal Hall effect. We calculated the optical Hall conductivity spectra $\sigma_{xy}(\omega)$ representing the normal Hall effect using Eq. (S1) at 0.49 T (blue curves). As compared to the experimental $\sigma_{xy}{}^{T}(\omega)$ (red curves) and $\sigma_{xy}{}^{M}(\omega)$ (black curves), the contribution from the normal Hall effect (Drude response) can be neglected above 40 meV.

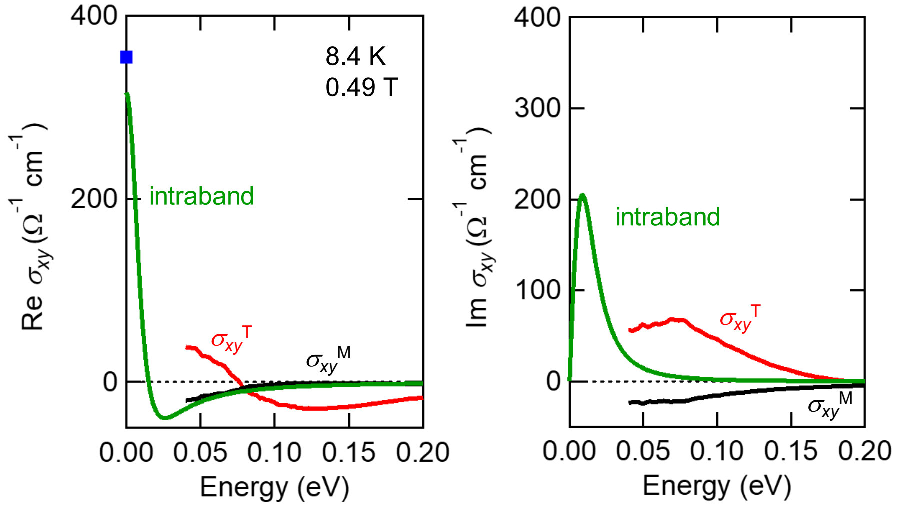

Supplementary Figure 7| The calculated intraband contribution. Topological optical Hall conductivity spectra arising from the real-space Berry phase (green curves). The blue square at zero energy represents the observed d.c. value of the σ_{xy}^{T}. For comparison, the experimental $\sigma_{xy}^{T}(\omega)$ (red curves) and $\sigma_{xy}^{M}(\omega)$ (black curves) dominated by the interband transition are also shown.

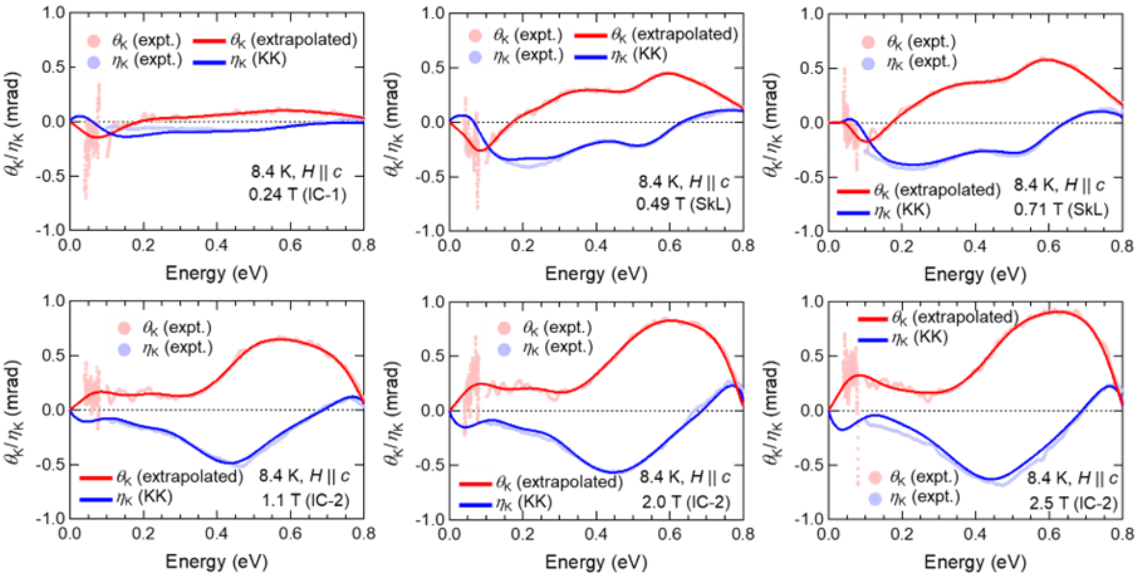

Supplementary Figure 8| Kramers-Kronig analysis of magneto-optical spectra at 8.4 K, for several magnetic fields. The light red and light blue markers indicate the independently measured Kerr rotation angle θ_K and ellipticity η_K, respectively. The red solid curves show the extrapolated θ_K after smoothing, and the blue solid curves show η_K deduced by a Kramers-Kronig transformation of the extrapolated Kerr rotation angle spectra θ_K.

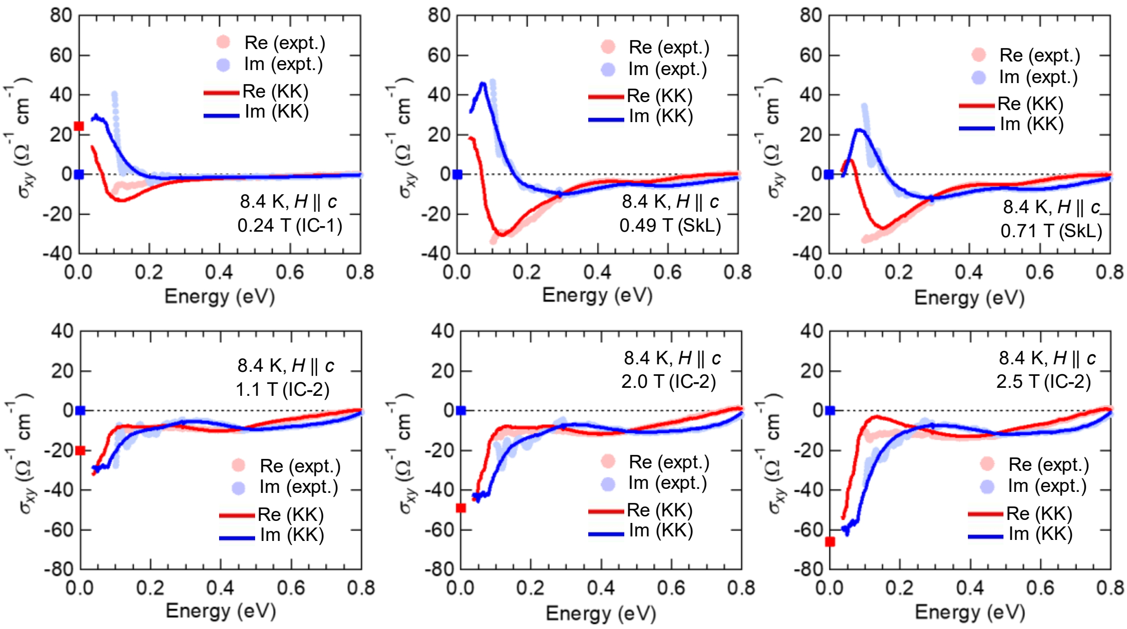

Supplementary Figure 9| Optical Hall conductivity spectra at 8.4 K for several magnetic fields. The light red and blue markers indicate the real and imaginary part of the optical Hall conductivity spectra, calculated from the independently measured θ_K and η_K, respectively. The red and blue solid curves respectively show the real and imaginary part of the optical Hall conductivity spectra, calculated with η_K spectra deduced from the Kramers-Kronig analysis of θ_K. The red and blue squares at zero energy represent the d.c. Hall conductivities acquired from transport measurement.

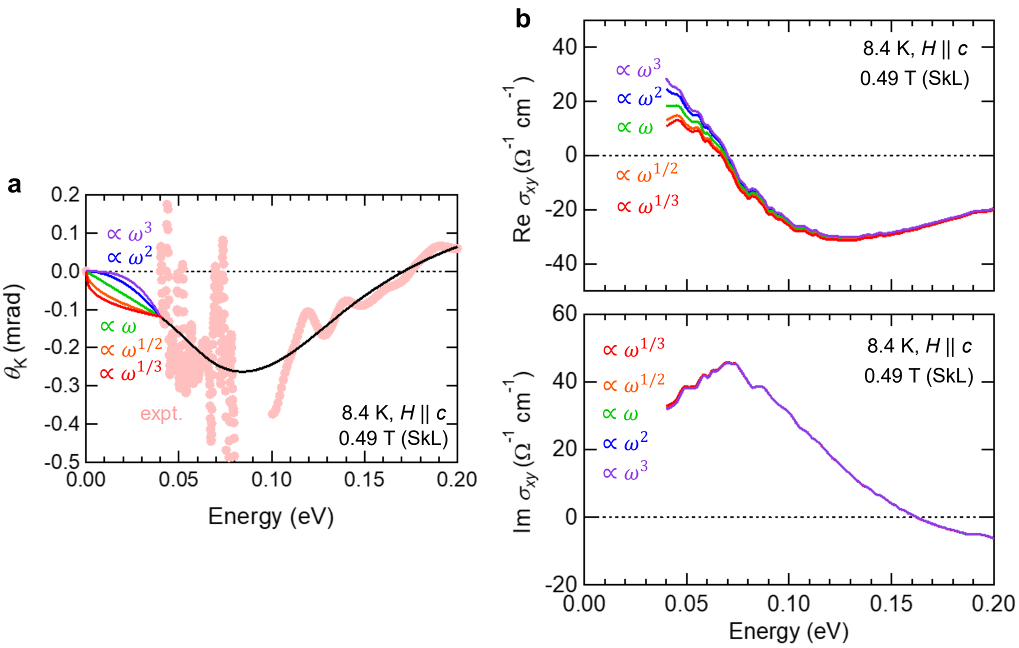

Supplementary Figure 10| The test of extrapolation functions for Kramers-Kronig analysis. a, The Kerr rotation angle θ_K with several extrapolations below 40 meV. The red, orange, green, blue, and purple curves show the extrapolations described by ~ $\omega^{1/3}$, $\omega^{1/2}$, ω, ω^2, and ω^3, respectively. The light red markers represent the experimental θ_K, and the black curve represents θ_K above 40 meV fitted by using polynomial functions. **b**, The real (upper panel) and imaginary (lower panel) parts of $\sigma_{xy}(\omega)$ deduced for each extrapolation function.

Supplementary References

1. Hirschberger, M. *et al.* Topological Nernst effect of the two-dimensional skyrmion lattice. *Phys. Rev. Lett.* **125**, 076602 (2020).
2. Kurumaji, T. *et al.* Skyrmion lattice with a giant topological Hall effect in a frustrated triangular-lattice magnet. *Science* **365**, 914–918 (2019).
3. Hirschberger, M. *et al.* High-field depinned phase and planar Hall effect in the skyrmion host Gd_2PdSi_3. *Phys. Rev. B* **101**, 220401 (2020).
